# A recurrent somatic missense mutation in *GNAS* gene identified in familial thyroid follicular cell carcinomas in German longhaired pointer dogs

**DOI:** 10.1186/s12864-022-08885-y

**Published:** 2022-09-23

**Authors:** Yun Yu, Freek Manders, Guy C. M. Grinwis, Martien A. M. Groenen, Richard P. M. A. Crooijmans

**Affiliations:** 1 Animal Breeding and Genomics, Wageningen University & Research, Droevendaalsesteeg 1, 6708 PB Wageningen, The Netherlands; 2 Princess Máxima Center for Pediatric Oncology, Heidelberglaan 25, 3584CS Utrecht, The Netherlands; 3 Department of Biomolecular Health Sciences, Division of Pathology, Faculty of Veterinary Medicine, Utrecht University, Yalelaan 1, Utrecht, The Netherlands

**Keywords:** Familial cancer, Thyroid carcinoma, Driver mutation, Dog, GNAS, Mutational signature, Whole genome sequencing

## Abstract

**Background:**

We previously reported a familial thyroid follicular cell carcinoma (FCC) in a large number of Dutch German longhaired pointers and identified two deleterious germline mutations in the *TPO* gene associated with disease predisposition. However, the somatic mutation profile of the FCC in dogs has not been investigated at a genome-wide scale.

**Results:**

Herein, we comprehensively investigated the somatic mutations that potentially contribute to the inherited tumor formation and progression using high depth whole-genome sequencing. A *GNAS* p.A204D missense mutation was identified in 4 out of 7 FCC tumors by whole-genome sequencing and in 20 out of 32 dogs’ tumors by targeted sequencing. In contrast to this, in the human TC, mutations in *GNAS* gene have lower prevalence. Meanwhile, the homologous somatic mutation in humans has not been reported. These findings suggest a difference in the somatic mutation landscape between TC in these dogs and human TC. Moreover, tumors with the *GNAS* p.A204D mutation had a significantly lower somatic mutation burden in these dogs. Somatic structural variant and copy number alterations were also investigated, but no potential driver event was identified.

**Conclusion:**

This study provides novel insight in the molecular mechanism of thyroid carcinoma development in dogs. German longhaired pointers carrying *GNAS* mutations in the tumor may be used as a disease model for the development and testing of novel therapies to kill the tumor with somatic mutations in the *GNAS* gene.

**Supplementary Information:**

The online version contains supplementary material available at 10.1186/s12864-022-08885-y.

## Background

We previously reported familial thyroid follicular cell carcinomas (FCCs) in 54 Dutch German longhaired pointers (GLPs), identified by histological examination and an additional 29 dogs were suspected to be affected based on typical clinical signs [1]. The familial FCC was heterogeneous with 5 different histological subtypes: follicular thyroid carcinoma (FTC), papillary thyroid carcinoma (PTC), compact thyroid carcinoma (CTC), follicular-compact thyroid carcinoma (FCTC), and carcinosarcoma. Two homozygous deleterious mutations in the *TPO* gene were identified to be the germline risk factors of FCC predisposition in these dogs, based on a genome-wide association study (GWAS) and whole-genome sequencing (WGS) analysis [2]. However, besides the germline risk factors, key somatic mutations (driver mutations) also play an important role. These mutations can lead to uncontrolled cell division, escape from apoptosis, immune evasion and accelerated tumour growth [3, 4]. Identifying these driver mutations can contribute to unraveling the molecular mechanism of tumorigenesis.

Canine FCC is, in morphology, highly similar to human thyroid carcinomas originating from follicular cells. The GLP dogs with FCC could be an important disease model for thyroid cancer (TC) research and therapy development in dogs and humans. The somatic mutation landscapes of follicular cell thyroid carcinoma in humans have been extensively investigated [5–7]. In human thyroid cancer, the *BRAF* p.V600E somatic mutation is the most common driver mutation. Other frequently identified mutations in human thyroid cancers are within the *RET, PTEN*, and the *RAS* gene family (*KRAS*, *NRAS* and *HRAS*) [8]. In contrast to humans, the somatic mutation landscape of TC in dogs is still unclear. Other studies have identified genes with somatic mutations in a variety of canine tumors with known gene roles in human cancers. Examples of the driver genes in tumorigenesis present in both species are: the homologous mutation to the human somatic *BRAF* p.V600E mutation in naturally occurring canine bladder cancer [9], the *FBXW7* mutation in lymphomas [10], the recurrent somatic *SETD2* mutation in osteosarcomas [11], the somatic *TP53* and *PIK3CA* mutations in multiple cancers [12], the somatic mutations in *TP53*, *PDGFRA*, *PIK3CA*, *EGFR* and *IDH1* in sporadic gliomas [13], and the *NRAS*, *FAT4*, *PTEN* and *TP53* mutations in melanomas [14].

In this study, we generated whole genome sequencing data from both the tumor tissue and the matched animal genome. The 7 animals included in this study were closely related and are homozygous for the *TPO* variants associated with the disease we reported in our previous study [2]. Somatic single nucleotide variants (SNVs), structural variants (SVs), and copy number alterations (CNAs) were investigated. The somatic mutation landscape was further investigated, including somatic mutational burden, driver genes or significantly mutated genes and mutational signatures. Meanwhile, several tumor tissue characteristics were also investigated, including purity, ploidy, telomere length and subclone cluster. Most interestingly, we identified a recurrent missense mutation in the *GNAS* gene, which is a novel somatic mutation and correlates with a lower tumor mutational burden. Unveiling the somatic mutations, along with previous identification of germline risk factor in the *TPO* gene, reveals the genetic bases of this familial FCC, which could help us to understand the tumor development and enhance the use of these dogs as a disease model.

## Results

### WGS and somatic mutation landscape

**Fig. 1:**
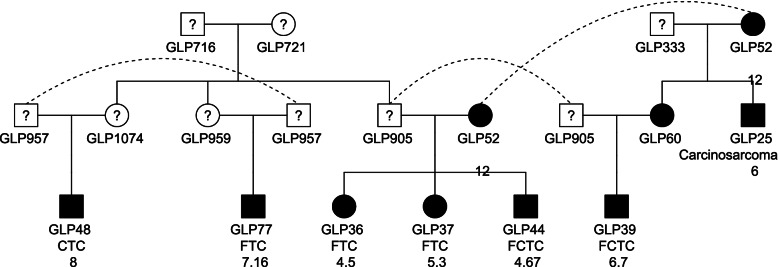
The pedigree of the 7 familial FCCs in this study. A circle and square denotes a female and a male dog respectively. Solid black indicates that the dog was affected. A question mark indicates that the disease status of the dog is unknown. The 3 rows of texts below a circle and square denote ID, thyroid cancer subtype, and age at diagnosis respectively. A dotted line indicates an identical dog

Whole-genome sequencing was performed on both tumor (FCC tissues) and matched normal (derived from blood DNA) samples from 7 GLP dogs. Additionally, RNA-seq was performed on each tumor sample. The 7 GLPs are closely related (Fig. 1). GLP36, 37 and 44 are full siblings where GLP48 and GLP77 are half siblings. GLP39 is the nephew of GLP25, and half-sibling of GLP36, 37 and 44.

The age at diagnosis of the FCC ranged between 4.5 and 8 years (Table 1). Five dogs were males and two were females (GLP 36 and 37). All 7 dogs were homozygous for the mutations in the *TPO* gene associated with the disease identified in our previous study [2]. The histological subtypes of FCCs include 3 FTCs, 2 FCTCs, 1 CTC, and 1 carcinosarcoma. The familial FCCs of these 7 dogs were heterogeneous in histology but were supposed to result from the same germline genetic risk factor.

**Table 1:** Sample information

Animal ID	Subtype	Age at diagnosis (years)	Sex ^a^	DogWUR ID—tumor (coverage)	DogWUR ID – normal (coverage)
GLP77	L:FTC; R:Adenoma	7.2	M	108 (68x)	115 (32x)
GLP48	L:CTC; R:FTC	8.0	M	109 (81x)	116 (18x)
GLP36	L:FTC; R:FCTC with bone	4.5	FS	110 (65x)	117 (17x)
GLP37	L:FTC; R:FTC	5.3	FS	111 (72x)	118 (20x)
GLP44	L:FCTC; R:FCTC	4.7	MC	112 (68x)	119 (35x)
GLP25	L:Carcinosarcoma; R:FCTC	6.0	M	113 (83x)	91 (32x)
GLP39	L:FCTC; R:FCTC	6.7	M	114 (75x)	120 (42x)

*Note*: ^a^M denotes male, FS denotes female spayed, MC denotes male castrated

### Somatic mutation burden

We identified 10,216 somatic SNVs, 1,034 small insertions, and 1,558 small deletions from the WGSs of the 7 GLPs, using our consensus calling method. On average, there were 10 (2—15) somatic mutations per sample that modified a protein, most of which were missense mutations (supplementary Figure S3A). The true positive calling rate among the somatic SNVs and Indels was estimated to be 0.83 by visual inspection. Furthermore, somatic SNVs have a higher true positive rate than Indels (0.90 > 0.57), similar to previous findings [15]. Transition to transversion ratio was between 0.91 and 1.81, with an average of 1.23 (supplementary Figure S3B).

Based on WGS, the average tumor mutation burden (TMB) of SNVs was estimated to be 0.58 (ranges 0.05—1.15) (mutations per megabase), which was estimated by the number of somatic mutations divided by the total length of the canine genome (2,500 Mb). The correlation between diagnosis age and tumor TMB was not significant (R^2^ = 0.04, p-value = 0.68, pearson correlation) (Supplementary Figure S4).

**Fig. 2:**
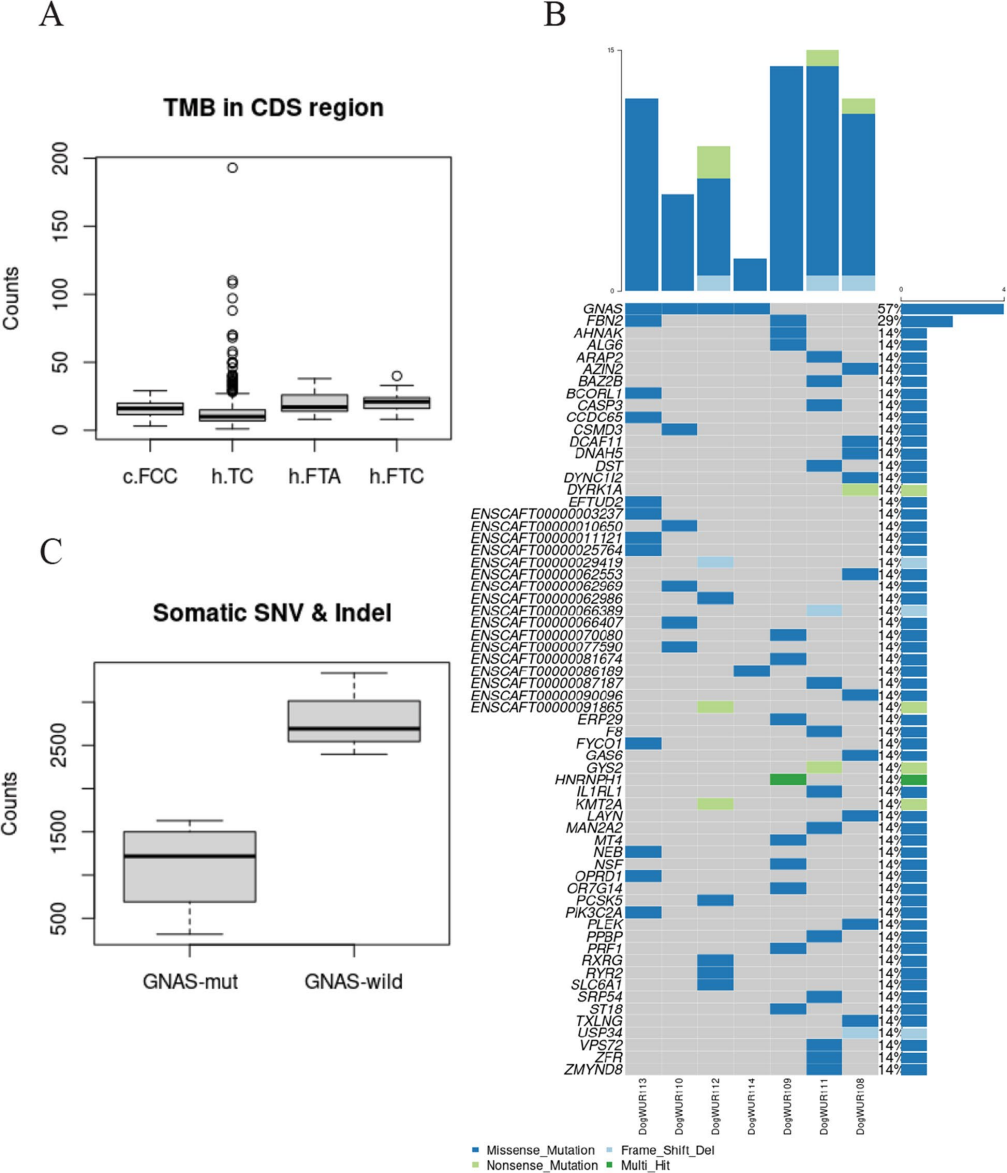
A. comparison of number of mutations identified in CDS region between canine tumors and 3 types of human thyroid tumors. Abbreviations: c.FCC – canine follicular cell carcinoma, h.TC – human papillary thyroid carcinoma (from TCGA project), h.FTA – human follicular thyroid adenoma, h.FTC – human follicular thyroid carcinoma. B. Mutation landscape of the 7 tumors. Each column represents one sample, each row represents one somatic mutated gene. The right bar chart represents the frequencies of gene alterations across the 7 tumors. The upper bar chart represents the number of mutation in exons across the 7 tumors. C. Number of somatic SNVs and Indels identified in 4 tumors with the GNAS p.A204D mutation (GNAS-mut) and 3 samples without that somatic mutation (GNAS-wild)

We calculated the number of mutations that occurred in coding regions (CDS) for all 7 tumors and compared them to the human thyroid cancer (THCA) data from The Cancer Genome Atlas (TCGA) program, and two other types of huamn thyroid cancer, follicular thyroid adenoma (FTA) and follicular thyroid carcinoma (FTC), from study of Jung et al. 2016 [16]. We found no significant difference between humans and dogs for these tumor types (Fig. 2A) (Wilcoxon rank sum test between dog FCC and human THCA: 0.1383, Welch t-test between dog FCC and human FTA: 0.3910, Welch t-test between dog FCC and human FTC: 0.1916).

### Recurrent events

*GNAS* and *HNRNPH1* were identified as being significantly mutated genes (SMG) by MuSiC2. The *GNAS* gene was also identified by the dNdScv algorithm. There was only one missense mutation p.A204D (chr24:43657087C > A) in the *GNAS* gene (exon 9), which was identified in 4 of 7 tumor samples (Fig. 2B). This mutation in the DogWUR110 sample (GLP36) was not identified by our consensus calling method but was rescued by the visual inspection. The variant allele frequency of the *GNAS* mutation was 0.28 on average (0.43 in GLP36, 0.22 in GLP44, 0.33 in GLP25, 0.14 in GLP39). The RNA-seq data also supported the *GNAS* mutation in those 4 dogs. The somatic mutations identified in the *HNRNPH1* gene turned out to be false positive calls after visual inspection.

Although these familial FCCs have the same germline susceptibility, the somatic mutation landscape seems to be heterogeneous. The somatic mutation in the *GNAS* gene presented in 4 out of the 7 GLPs. The other 3 dogs (GLP37, 48, 77) had unclear driver events, suggesting heterogeneity of driver mutations among these samples. Driver mutations frequently identified in humans, such as mutations in the genes *BRAF*, *RAS*, *TP53*, *PTEN*, were not observed in the dogs used in this study (Fig. 2B). Somatic structural variants and copy number alterations were also investigated, but no driver gene was identified from them (details in supplementary).

Canine *GNAS* p.A204 corresponds to human *GNAS* p.A249 (protein accession: P63092). The homologous mutation in humans at chr20:58,909,711 has not been reported in human dbSNP [17]. Canine *GNAS* p.A204D was predicted to be possibly damaging with a value of 0.552 by PolyPhen-2, and deleterious by PROVEAN with a score of -5.460. The *GNAS* p.A204 was conserved across species with a conservation score of 8 (range 1–9) according to estimation of ConSurf. Furthermore, the amino acid A (Ala) is non-polar while D (Asp) is polar and hydrophobic with a negative charge. These observations suggest that the mutation may have a big impact on the function of the GNAS protein. Canine *GNAS* p.A204 is a novel mutation in dogs, not reported either in dbSNP nor in the 722 dog genome panel.

Interestingly, we found that the tumors with the *GNAS* mutation (GNAS-mut) had significantly less somatic SNVs and Indels compared to tumors without the *GNAS* mutation (GNAS-wild) (Welch t-test, *p*-value 0.0082) (Fig. 2C), likewise they also contained less protein-modifying somatic mutations.

To identify the molecular signaling pathways promoting tumorigenesis, we performed a gene expression differentiation analysis contrasting tumors with and without the somatic *GNAS* mutation. PCA analysis didn’t identify a clear distinction between GNAS-mut and GNAS-wild samples (Supplementary Figure S5A). Moreover, there was no difference in expression level of the *GNAS* gene between these two groups (Supplementary Figure S5B). Differential gene expression analysis contrasting the 4 GNAS-mut samples and 3 GNAS-wild samples identified 53 differentially expressed genes by a significant threshold of 0.05 adjusted p-value. But no enriched biological process or KEGG pathway was identified by pathway enrichment analysis based on these 53 differentially expressed genes. This suggests that there is probably no difference in the molecular signaling pathways that contribute to the tumorigenesis between these two groups of dogs.

### Association with morphological characteristics

*GNAS* mutation might be associated with an increased proliferation rate according to semiquantitative evaluation of the number of mitotic figures in the neoplasms. However, additional, quantitative analysis including the use of a proliferation marker such as Ki67 is necessary to determine whether or not there is a relationship between proliferation and the *GNAS* mutation. Other histological characteristics appear not to be associated with the mutation.

### Prevalence of the *GNAS* mutation

To identify the prevalence of the *GNAS* p.A204D somatic mutation among dogs suffering from FCC, we genotyped 49 tumor samples from 34 affected dogs using Sanger sequencing (supplementary Table S1). Of 13 dogs, tumor tissue from both the left and right thyroid gland was genotyped successfully. However, genotyping failed in 4 samples from 4 dogs. Finally, we obtained 45 genotypes covering 32 GLPs. We found that tumors from 20 of the 32 affected dogs had this *GNAS* somatic mutation, resulting in a prevalence of 62.5%. We also performed Sanger sequencing on normal DNA (obtained from blood) of the 7 dogs used in this study and none of them captured the *GNAS* mutation, confirming that these were somatic mutations. To ensure further that the *GNAS* p.A204D mutation is generally not present as a germline variant, we checked that this mutation was not present in the whole genome sequences that we previously obtained from blood DNA of 22 GLPs [2].

The germline genotype of the marker in the *TPO* gene associated with FCC was available for 31 dogs (supplementary Table S1). We tested whether the *TPO* germline mutation and *GNAS* somatic mutation were significantly correlated, but this was not the case (Chi-square test; *p*-value = 0.237).

### Telomere length

**Fig. 3:**
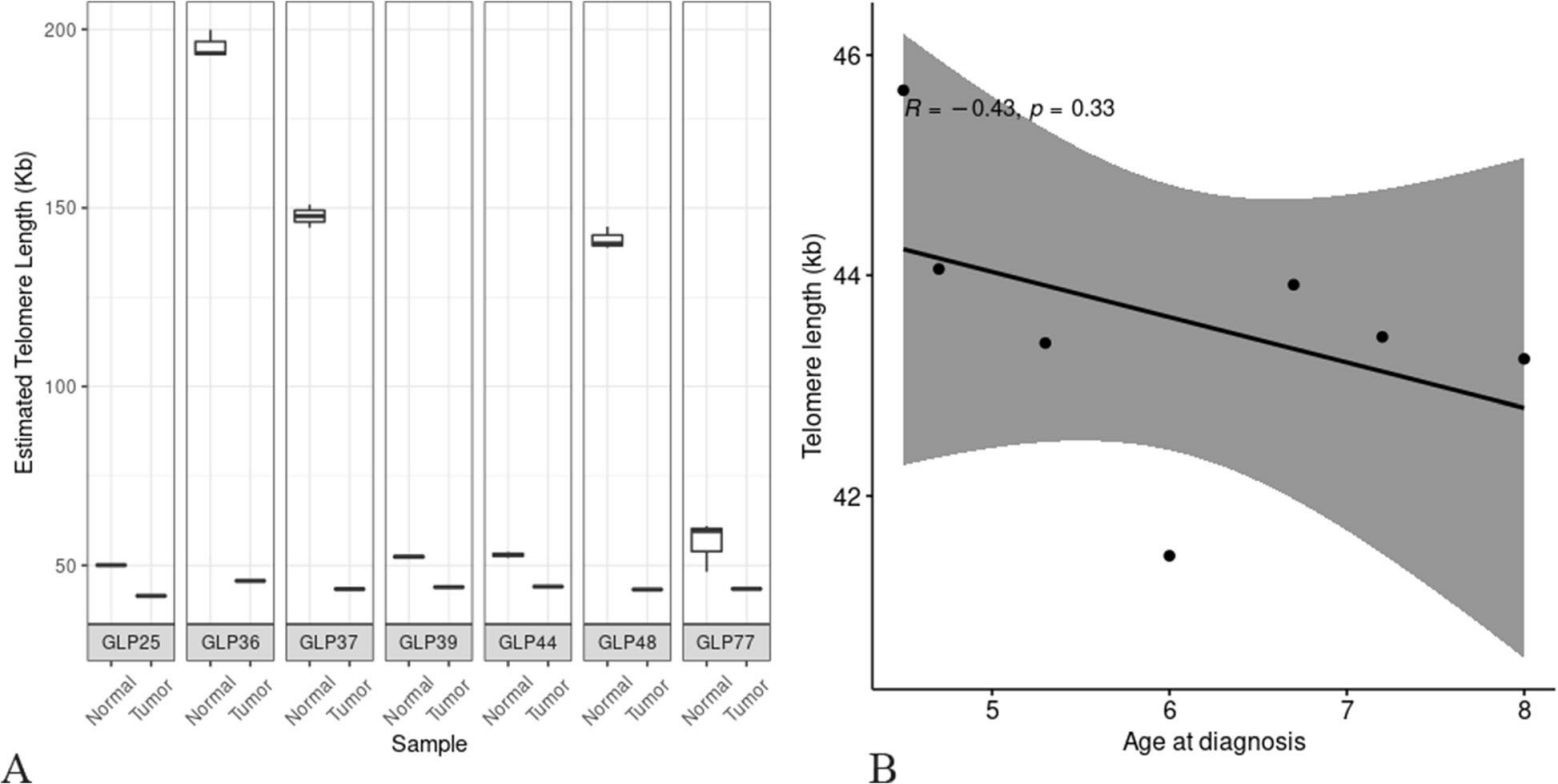
A. Boxplot of telomere length estimated from WGS of each tumor and matched normal sample. B. Correlation between the tumor telomere length and diagnosis age of FCC (years). The shaded area represent 95% confidence interval

Tumor genomes have significantly shorter telomeres compared to normal genomes according to our estimation using TelSeq (Fig. 3A), which is in concordance with our knowledge about telomere shrinkage in tumor cells [18]. There is no significant correlation between telomere length and diagnosis age of FCC (Fig. 3B). To maintain the length of the telomeres in the tumor cells, telomerase is often activated. While in these 7 tumor samples, *TERT* expression was only detected in one dog (GLP25; DogWUR113). Additionally, somatic mutations in the *TERT* gene or its’ promoter regions were not identified. Therefore, telomerase was assumed not to be activated in these tumors.

### Tumor purity, ploidy and subclone cluster

To explore the intra-tumor heterogeneity of FCC in these GLPs, we identified the subclone cluster in the 7 tumors along with purity and ploidy. According to the estimation from the TitanCNA workflow, 2 tumor samples had a good tumor cell purity of above 0.5, while 5 samples had relatively low purity, ranging between 0.3 and 0.4 (Table 2). Ploidy was estimated to be between 2 and 3 (Table 2). A subclone cluster was identified in only one tumor sample, DogWUR108, where the cancer cell fraction of the subclone was estimated to be 0.46. These tumors were supposed to have a low intra-tumor heterogenity based on the subclone identification, which is also consistent with our previous expectation about stage from our clinical and histological examination where most tumors were supposed to be low grades.

**Table 2:** Purity and ploidy estimated for each tumor sample

	DogWUR108	DogWUR109	DogWUR110	DogWUR111	DogWUR112	DogWUR113	DogWUR114
Purity	0.78	0.56	0.38	0.34	0.35	0.33	0.32
Ploidy	2.2	2.9	2.6	2.2	2.6	2.5	2.1

### Mutational signatures

**Fig. 4:**
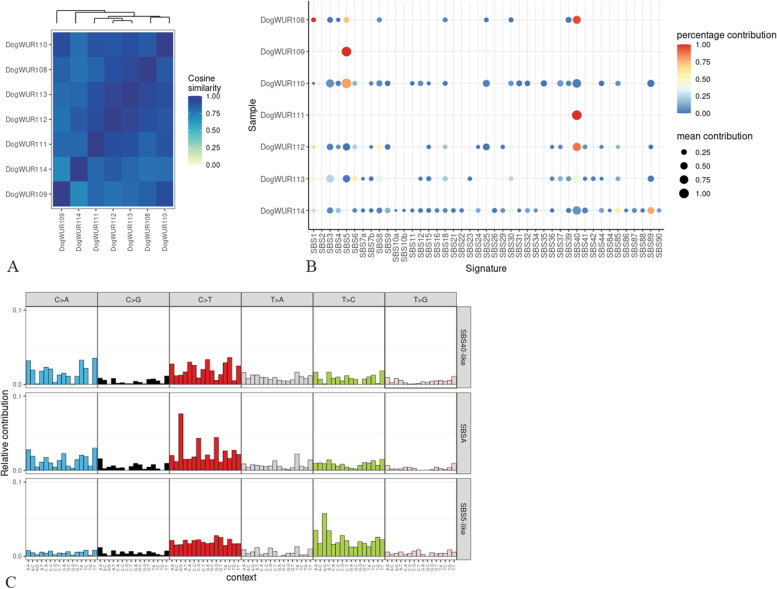
A. Cosine similarity of the somatic mutational spectrum across the 7 tumors. B. Contribution of known human mutational signatures to somatic mutations identified in each tumor using bootstrapping refitting in 1000 iterations. The color of the dot shows the percentage of iterations in which the signature is found (contribution > 0). The size of the dot represents the average contribution of that signature (in the iterations in which the contribution was higher than 0). C. Mutational signatures extracted from the 7 tumors using nonnegative matrix factorization method

A mutational signature is the outcome of a mutagenic process comprising some form of DNA damage, subsequently acted upon by DNA repair and/or replicative machinery. Mutational signatures can reveal potential sources of mutagenesis during tumorigenesis [19]. For this, we explored the substitution mutational signatures. The mutational spectrum of the somatic substitutions is highly similar across these 7 tumors, based on their cosine similarity (Fig. 4A). We fitted the existing COSMIC signatures to our mutational spectrum [20]. Bootstrapping was used to increase the confidence of our results. We identified that SBS1, SBS5 and SBS40 contributed most to the mutation profile (Fig. 4B). Next, to signature refitting, three mutational signatures were extracted from somatic SNVs identified from the 7 tumors using nonnegative matrix factorization method, of which two were highly similar to known human signatures SBS40 and SBS5, and another mutational signature SBSA was most similar to SBS1 (similarity of 0.71) (Fig. 4C). This is consistent with the results from the bootstrapping refitting. SBS5 was also the most frequently identified SBS in human THCA [7, 21]. SBS1 and SBS5 are clock-wise signatures. SBS40 also correlates with patients’ ages for some types of human cancers. Moreover, C > T transitions in CpGs were the most common point alterations and correlate with age in human cancers. Enrichment of these mutations suggests the role of age in the somatic mutation accumulation and tumorigenesis and also indicates that the source of mutagenesis was endogenous in these dogs.

## Discussion

In this study, we performed comprehensive genomic analyses of familial FCCs in Dutch German longhaired pointers using whole-genome sequencing and RNA-seq data. Our somatic mutation profiling of the tumors identified a somatic missense mutation in the *GNAS* gene, which is present in 4 of 7 tumor WGS samples and 20 of 32 tumors of GLPs genotyped by sanger sequencing. It is a novel mutation identified in dogs and its’ homologous mutation in humans has not been reported. Therefore, it is very likely a novel driver mutation.

The GNAS protein, also known as the alpha-subunit of the stimulatory G protein (Gαs), normally activates adenylyl cyclase downstream of activated G-protein-coupled receptors (GPCRs), in response to hormones and a diverse number of extracellular signals. This results in the generation of a second messenger called cAMP, which activates protein kinase A (PKA). Activated PKA can phosphorylate downstream targets that are involved in many pathways and evoke downstream signaling cascades. The *GNAS* gene is included as a tier 1 proto-oncogene in the cancer gene census (CGC) database. The mutations in genes involved in this GPCR pathway were identified in many different types of cancers, including lung adenocarcinoma and breast cancer [22]. The link between the *GNAS* mutation and tumorigenesis has been proven. The marker in the *GNAS* gene has also been included in a diagnostic panel of thyroid cancer (ThyroSeq) [23]. The activating mutations in the *GNAS* gene can result in overactivation of thyroid stimulating hormone receptor (TSHR) signaling and accumulation of cellular cAMP. The accumulation of cAMP in thyrocytes can lead to uncontrolled cell proliferation [24]. Interestingly, semiquantitative assessment of 36 FCCs in the study population suggests a possible increase in mitotic rate associated with *GNAS* p.A204D mutation identified in this study. However, in order to obtain more robust data on such association, more thorough, quantitative analysis of the proliferation rate of the neoplastic cells using a proliferation marker like Ki67 is necessary because the number of mitotic figures typically underestimates the actual percentage of proliferating neoplastic cells. Moreover, elevated *GNAS* expression can enhance cancer cell migration [25]. In contrast, *GNAS* knockout mice have shown reduced beta cell proliferation [26].

*GNAS* mutations often lead to benign thyroid cancer [27], but can also be found in malignant thyroid cancer [24]. In humans, *GNAS* mutations were also proposed to be markers of benign nodules. Interestingly, in these dogs, tumors with *GNAS* p.A204D mutation had lower amounts of somatic mutations (SNV, Indel, and SV). A high tumor mutational burden (TMB) usually correlates with poor survival outcomes in humans [28, 29]. We thus suspect that patients with the *GNAS* mutation may have a better prognosis. However, we don’t have survival data to validate this. This needs to be investigated further. The impact of this mutation on the function of the GNAS and downstream signaling pathways is not clear. Although we performed differentially expressed gene analysis and pathway enrichment analysis based on RNA-seq data, no enriched biological process term or KEGG pathway was found. How this mutation impacts the downstream signaling pathway, such as the cAMP level, was not investigated in this study. Further experiments are needed to reveal the influence of this *GNAS* mutation on the cellular cAMP level and downstream signaling pathways.

According to studies on the somatic mutation landscape in canine cancers, sporadic canine cancers have similar driver genes to corresponding human cancers [9–14]. In a study screening 33 canine cancer cell lines, driver genes similar to human cancers were also identified [30]. However, thyroid cancer seems to be an exception. It has different somatic mutation landscape between dogs and humans.

Campos et al., investigated the somatic mutation landscapes of 43 canine FCCs and 16 canine MTCs by targeted sequencing of *HRAS*, *NRAS*, *PIK3CA*, *BRAF*, *RET*, and *PTEN* genes [31]. They identified 2 missense mutations in the *KRAS* gene which have also been reported in TC of humans with a similar prevalence. No missense mutations were found in the sequenced regions of *HRAS*, *NRAS*, *BRAF*, *PIK3CA*, and *RET* nor in the entire coding sequence of *PTEN*. Hence, the mutations most commonly involved in thyroid tumorigenesis in humans were thought to be rare and not to play a major role in thyroid tumorigenesis in dogs. Unfortunately, the *GNAS* mutation was not investigated in that study, thus the prevalence of the *GNAS* mutation in their affected dogs is unknown.

In human thyroid cancer, driver mutations in the *GNAS* gene were also identified, but usually at a low prevalence [6, 8, 24, 32]. In 492 human thyroid carcinoma samples from the TCGA, mutations in the *GNAS* gene were detected in only 2 patients and these 2 mutations in the *GNAS* gene were at different locations from *GNAS* p.A204D [8]. In the COSMIC database, 73 of 3724 thyroid tumors capture mutations in the *GNAS* gene, but all at different locations. In a study including 65 human FTC samples, genetic alterations in *GNAS* were found in 5 of them (R201H in 3 samples) [6]. In another study with 154 hot thyroid nodules, 10 samples capture mutations in the *GNAS* gene [32]. The prevalence of *GNAS* mutations in TC ranges from 0.22%—2.12% according to an investigation in 1841 human thyroid tumors [24].

In our dogs with a familial FCC, the *GNAS* mutation was the most common somatic mutation identified (20 out of 32 affected dogs). The prevalence of this mutation in the affected dogs may be even higher because by chance some samples used for sequencing may contain only healthy cells but no tumor cells, resulting in false negatives. The difference in the prevalence of *GNAS* mutations in humans and our dog samples could suggest a species difference in the driver mutations for thyroid cancer between GLPs and humans. Furthermore, how this somatic mutation occurred and survived from DNA damage repair activity in that many affected dogs is also an interesting and important question. Further research is needed to answer these questions. We speculated that the germline risk factor for the FCC in some way may induce this *GNAS* mutation. We therefore tried to test the correlation between the germline risk factor in the *TPO* gene and the somatic *GNAS* mutation. However, the *GNAS* somatic mutation was also identified in the affected dogs that were supposed to be spontaneous FCC cases (3 out of 8 dogs) based on the genotype of the marker identified in the *TPO* gene. In all GLPs with the homozygous recessive genotype in the *TPO* gene, the *GNAS* somatic mutation was identified in 62.5% of them. The chi-squared test didn’t show a significant correlation between the germline *TPO* mutation and the *GNAS* somatic mutation. There seems to be an interaction between the germline mutation in the *TPO* gene and the somatic mutation in the *GNAS* gene but it is weak.

*GNAS* aberrations have been identified not only in thyroid tumors, but also in many other tumors in humans. Deep sequencing analysis has revealed that around 4.2% of tumors carry *GNAS* activating aberrations [33]. According to an investigation in 274,694 tumors in humans, the most common *GNAS* alterations were copy number variants (60.5%; all of which were amplifications), followed by GNAS codon R201 activating point mutations (34.8%). All other alterations (including the activating Q227 mutation) account for less than 5% [24]. The mutation p.R201C (in exon 8) is the most prevalent point mutation in *GNAS*, which was identified in many cancers, including thyroid cancer [34, 35]. The p.R201H mutation was also found in thyroid cancer [36].

In general, dogs have great potential as disease models of human cancers. Dogs are now increasingly highlighted as disease models for cancer research. In dogs, the report of *GNAS* mutations is still limited. According to the authors’ knowledge, there was only one report where *GNAS* mutations were identified in canine adrenocortical tumors [37]. Here, we identified a familial FCC with high prevalence of *GNAS* p.A204D mutation in Dutch GLPs. Together with the previously identified germline risk factor in the *TPO* gene, the genetic basis of the TC development in these dogs is becoming increasingly clear. These dogs could be developed as a disease model for research and translational medication trials for thyroid and possibly other tumour types, with somatic mutations in the *GNAS* gene.

The current study has some limitations. The relatively small sample size limited the power of our analyses to identify recurrent somatic SVs and CNAs. A larger sample size may help to identify the recurrent somatic SV and CNA event in the future to elucidate whether somatic SVs or CNAs also contribute to this familial FCC. Although *GNAS* mutations were proven to be able to affect downstream signaling pathways, the potential effect of *GNAS* mutation p.A204D identified here was not further investigated. Further experiments are needed to elucidate how this mutation leads to tumorigenesis.

## Conclusions

In this study, we profiled somatic mutation landscape of the FCC in GLP dogs at a genome-wide scale and identified a promising novel recurrent mutation of it, the *GNAS* p.A204D mutation. The prevalence of somatic mutation in the *GNAS* gene in TC is different between our dogs and humans. Our findings provide novel insights in potential molecular mechanism of thyroid carcinoma development. Moreover, our dogs with the FCC might be used as a good disease model for tumors with driver mutation in the *GNAS* gene.

## Materials and methods

### Samples

We selected 7 GLPs affected by familial FCC from the dataset described previously, and inclusion in this study was approved by the owners [1]. Tumor tissues and blood samples were collected during the surgery or necropsy. The tumor tissue for genetic testing was preserved in RNA-later (RNA stabilization reagent: Qiagen, Hilden, Germany) and blood was collected in K3-EDTA tubes. For histopathology, tumor tissue was fixed in 10% neutral-buffered formalin. All tumors of these dogs were evaluated histopathologically (Table 1). In order to correlate histomorphological characteristics of the neoplasms with mutations, other than histological pattern, the tumors were assessed semiquantitatively for several parameters (i.e. pleomorphism, anaplasia, necrosis, number of mitotic figures, infiltrative growth, aspect of cytoplasm and immunohistochemistry for thyroglobulin).

### Whole genome sequencing

DNA from blood was extracted using the Gentra Puregene Blood Kit (Qiagen, Hilden, Germany). The DNA from the tumor tissue stored in RNAlater reagent was extracted using Nucleospin Tissue kit (Bioke, Leiden, Netherlands). Library construction and sequencing are described in supplementary.

The tumor tissue of the left thyroid gland was sequenced to a depth of 60x. The genome of four dogs was sequenced with a coverage of 30 × whereas three dogs were sequenced with a coverage of 10x. The program FastQC [38] was used to evaluate the quality of sequencing. Sickle [39] was used to trim the reads using default settings. Sequences were aligned to the CanFam 3.1 reference genome downloaded from Ensembl following the best practice guideline (https://gatk.broadinstitute.org/hc/en-us/articles/360035535912-Data-pre-processing-for-variant-discovery). Mapping was done using BWA-MEM algorithm (current version 0.7.15) [40], followed by sorting with samtools 1.9 [41] and marking of duplications with Picard tool [42]. Finally, base quality score recalibration was performed using GATK 4.1.8.1 [43].

### RNA-seq

RNA-seq of tumor tissue was obtained and mapped to dog reference genome CanFam3.1, as described previously [2]. Mapping was performed using HISAT2 [44]. FeatureCounts [45] was used to quantify mapped reads to genomic features such as genes, exons, gene bodies, genomic bins and chromosomal locations. DESeq2 package [46] was used for differential expression analysis. The clusterProfiler package [47] was used to perform the gene set enrichment analysis.

### Somatic SNV & Indel

Three methods, Mutect2 [48], VarScan2 [49], and Strelka2 [50], were used to call the somatic SNVs in paired tumor-normal model. Firstly Mutect2 in tumor only model was run for each normal sample, folowed by GenomicsDBImport and CreateSomaticPanelOfNormals tools to create the panel of normal (PON) file. This PON file and the germline SNVs file obtained from study of Plassais et al., 2019 [51] using 722 dogs were incorporated in the somatic SNVs & Indels calling using the Mutect2 program in paired mode. The identified somatic variants were additionally filtered by CONTQ > 30, MBQ > 30, GERMQ > 30, MMQ > 30, VAF > 0.1, alternative allele count in normal sample = 0, alternative allele count > 5 in tumor sample, depth in tumor > 30, depth in normal sample > 8. The second method, VarScan2 paired mode, was run using the command: –tumor-purity 1 –min-coverage 8 –min-coverage-normal 6 –min-coverage-tumor 8 –min-reads 5 –min-avg-qual 30. Only the resulting high-confidence somatic variants were retained for subsequent analyses. The third model, Strelka2 paired mode, was run in default setting. Only variants passing the default filtering were used for the subsequent analyses. In addition, mutations with MQ < 30 were discarded.

A consensus approach was used to identify the reliable somatic SNVs and Indels. Bcftools (v1.9) [52] was used to intersect the variants identified by the 3 methods described above. Only the variants identified by at least two methods were considered as reliable and used for subsequent downstream analyses.

Visual inspection of somatic mutations in the genome browser Jbrowse [53], was used to detect false positive calls. We removed mutations identified in the simple repeat region and AG/TC tandem repeat regions. The simple repeat region file in correspondence with canFam3 was downloaded from the UCSC database. The AG/TC repeat regions were identified using BSgenome package [54] in R. The AG/TC repeat region was defined by 5 or more tandem AG/TC repeats. 5% Of all variants were randomly selected for visual inspection, to evaluate the true positive calling ratio among the final somatic SNVs and Indels dataset.

The VCF file containing somatic SNVs and Indels was transformed to mutation annotation format (MAF) file using vcf2maf [55] which depends on ensembl’s VEP tool. The maftools package [56] was used to analyze the somatic mutations, including generating the oncoplot, which shows the mutation landscape of samples, and the lolliplot, which shows the location of non-synonmous mutations in corresponding genes.

### Somatic copy number segmentation

Somatic copy numbers were called for paired tumor-normal samples using HMMCopy tool [57] (version 1.32.0) using the author’s recommendations. Briefly, GC counts and mappability files for CanFam3.1 reference genome were generated with 1000 bp window size using hmmcopy-util and GenMap [58] respectively. Read counts for each of tumor and normal bam files were generated in 1000 bp window size using readCounter in the HMMCopy package. GC counts, mappability and read counts were fed into the HMMCopy algorithm and segmentations were called using Viterbi algorithm. The segmented CNAs were fed to GISTIC2 (v2.0.22) [59] for identification of recurrent somatic CNAs. GISTIC2 identifies genomic regions that are significantly gained or lost across a set of tumors.

### Purity, ploidy estimation

TitanCNA [60] was used to estimate the purity and ploidy of tumors. Firstly, allele counts of tumors at heterozygous sites which overlapped with germline variants identified in 722 dogs [51] were generated using the Bcftools mpileup tool. Then the allele counts and somatic CNAs were fed into the TitanCNA algorithm to infer allele-specific copy numbers, copy-number based clonality, purity, ploidy, and cellular prevalence. Cellular prevalence is the proportion of tumor cells harbouring a somatic event.

### Significantly mutated genes

The MuSiC2 [61] and dNdScv [62] packages were used to identify the significantly mutated genes (SMG) from the somatic SNVs and InDels. MuSiC2 defined significantly mutated genes that have a significantly higher mutation ratio than the background somatic mutation burden across the tumor genomes. The dNdScv package identifies driver genes by quantifying the dN/dS ratios for missense, nonsense and essential splice mutations. Furthermore, ConSurf [63] was used to estimate the conservation score of identified amino acid changes in the SMG.

### Mutational signature

Single base substitutions (SBS) mutational signatures were constructed using the MutationalPatterns package [64]. A high true positive ratio of somatic SNVs identified of 90% make the mutational signatures analysis reliable. SBS was classified according to the 6 possible substitutions (C > A, C > G, C > T, T > A, T > C, T > G), plus the flanking 5’ and 3’ bases. Non-negative matrix factorization (NMF) was run with a rank of 2–7 and a final rank of 3 was chosen. The reconstructed mutational signatures were compared to known COSMIC mutational signatures detected in humans. A signature was considered novel when its similarity to other defined COSMIC mutational signatures was less than 0.85. Next, we used the “fit_to_signatures_strict” function to fit the COSMIC signatures to the mutation profiles. This function was used to reduce overfitting. The signatures that were present according to this refitting were then used to perform bootstrapped refitting, using 1000 iterations, to determine the confidence of the refit.

### Telomere length

Telomere length was estimated for each library using the tool TelSeq [65] along with a set of parameters to be compatible with our dataset: genome length of 2.5 Gb, 78 telomere ends, and reads length of 150 bp. The estimation of this tool has been shown to correlate well with Southern blot measurements based on 260 samples from the TwinsUK cohort [65].

### Validation

PCR was done using 60 ng of genomic DNA, with 0.4 µm of each primer and FIREPol 5 × Master Mix 7.5 mM Mastermix(Bio-Connect) in a final volume of 12 µl. Initial denaturation for 1 min at 95 °C was followed by 35 cycles of 95 °C for 30 s, 55 °C for 45 s, 72 °C 90 s, followed by a 5 min extension 72 °C. PCR primers for somatic mutation are GCACGTTTTGCTCTTTCGAT forward and TCCACAAACCTGTTGTTCCA reverse. After PCR the products were cleaned up with the use of Millipore PCR clean-up vacuum system (Multiscreen_PCR vacu 030, Merck Millipore). Sequencing reaction was done using 10 – 20 ng of cleaned PCR product, with 5 × dilution buffer, BigDye v3.1 reaction mix (Thermofisher) and 0.8 pmol/µl reverse primer. A sequencing reaction clean-up was performed with NaAc-EDTA and pure Ethanol. Sanger sequencing was performed on the ABI 3730 DNA sequencer (Applied Biosystems). SNP detection was performed using preGap and Gap (Staden Package).

**Table:** 

Software and algorithms	Version	Identifier
bcftools	v1.10.2	http://samtools.github.io/bcftools/bcftools.html
BSgenome	v1.58.0	https://bioconductor.org/packages/release/bioc/html/BSgenome.html
BWA	v0.7.15	https://github.com/lh3/bwa
clusterProfiler	v3.18.1	https://bioconductor.org/packages/release/bioc/html/clusterProfiler.html
ConSurf		https://consurf.tau.ac.il/
Delly	v0.8.3	https://github.com/dellytools/delly
DESeq2	v1.30.1	https://bioconductor.org/packages/release/bioc/html/DESeq2.html
dNdScv	v0.0.1.0	https://github.com/im3sanger/dndscv
Featurecounts	v2.0.1	http://subread.sourceforge.net/
GATK	v4.1.8.1	https://gatk.broadinstitute.org/hc/en-us
GenMap	v1.3.0	https://github.com/cpockrandt/genmap
GISTIC2	v2.0.22	https://www.genepattern.org/modules/docs/GISTIC_2.0
GRIDSS	v2.10.2	https://github.com/PapenfussLab/gridss
HISAT2	v2.2.0	http://daehwankimlab.github.io/hisat2/#:~:text=HISAT2%20is%20a%20fast%20and,to%20a%20single%20reference%20genome
HMMCopy	v1.32.0	https://bioconductor.org/packages/release/bioc/html/HMMcopy.html
karyoploteR	v1.16.0	http://bioconductor.org/packages/release/bioc/html/karyoploteR.html
maftools [47]	v2.6.05	https://bioconductor.org/packages/release/bioc/html/maftools.html
Manta	v1.6.0	https://github.com/Illumina/manta
MuSiC2	v0.2	https://github.com/ding-lab/MuSiC2
MutationalPatterns	v3.0.1	https://bioconductor.org/packages/release/bioc/html/MutationalPatterns.html
Mutect2—GATK4	v4.1.0.0	https://gatk.broadinstitute.org/hc/en-us
NMF	v0.23.0	https://cran.r-project.org/web/packages/NMF/index.html
picard	v2.23.8	https://broadinstitute.github.io/picard/
R	v4.0.3	https://cran.r-project.org/
Rstudio	v1.3.1093	https://www.rstudio.com/
Samplot	v1.3.0	https://github.com/ryanlayer/samplot
samtools	v1.9	http://www.htslib.org/
Strelka2	v2.9.2	https://github.com/Illumina/strelka
StructuralVariantAnnotation	v1.6.0	https://www.bioconductor.org/packages/release/bioc/html/StructuralVariantAnnotation.html
SURVIVOR	v1.0.7	https://github.com/fritzsedlazeck/SURVIVOR
SvABA	v1.1.3	https://github.com/walaj/svaba
TelSeq	v0.0.1	https://github.com/zd1/telseq
TitanCNA	v1.17.1	https://bioconductor.org/packages/release/bioc/html/TitanCNA.html
VarScan2	v2.4.4	http://varscan.sourceforge.net/
Vcf2maf	v1.6.18	https://github.com/mskcc/vcf2maf
VEP	v101.0	https://www.ensembl.org/info/docs/tools/vep/index.html

## Supplementary Information

**Additional file 1:** **Figure S1.** Landscape of somatic SVs identified in thetumors of 4 dogs. The circles represent deletion, insertion, inversion,duplication from the outside inwards respectively. The links inside representthe translocations. Different colors represent specific tumor sample, namelyred - DogWUR108, blue - DogWUR112, yellow - DogWUR113, green - DogWUR114. **Figure S2.** Recurrent copy number alteration identified byGISTIC2 in 4 dogs used in the analysis. A. Recurrent amplifications acrosscanine chromosome 1 - 38. A solid green line indicates the significancethreshold. B. Recurrent deletions across canine chromosome 1 - 38. A solidgreen line indicates significance threshold. **Figure S3.**  Summary of somatic SNVs and Indels identified in the 7 canine tumors. A.Summary of somatic SNVs and Indels derived from maftools package, includingvariant classification, variant type, SNV class, number of variants in codingregion, and top 10 mutated genes. B. Somatic SNVs substitution type andtransition and transversion. **Figure S4.** Correlation between age at diagnosis and tumormutation burden (mutation per Mb). **Figure S5.** A. PCA plot of the 7 tumors based on geneexpression. B. Expression level of the *GNAS* gene in tumors with andwithout somatic *GNAS* mutation. **Figure S6.** Coverage of RNA-seq reads mapped to *CNTN4*(A), *JAK3* (B), *CATSPERE* (C) gene for the 7 tumor samples. **Table S1.**  Genotypes of the somatic GNAS mutation and germline TPO mutation in dogs.

## Data Availability

The datasets generated and/or analysed during the current study are available in the EMBI-EBL ENA repository, https://www.ebi.ac.uk/ena/browser/search. Accession ID = PRJEB47059.
