# Supplementary material for: A recurrent somatic missense mutation in GNAS gene identified in familial thyroid follicular cell carcinomas in German longhaired pointer dogs
Source: BMC Genomics. 2022 Sep 23;23:669. doi: 10.1186/s12864-022-08885-y (PMC9508735; doi:10.1186/s12864-022-08885-y)
Supplement: Supplementary file 1 — Additional file 1: Figure S1. Landscape of somatic SVs identified in thetumors of 4 dogs. The circles represent deletion, insertion, inversion,duplication from the outside inwards respectively. The links inside representthe translocations. Different colors represent specific tumor sample, namelyred - DogWUR108, blue - DogWUR112, yellow - DogWUR113, green - DogWUR114. Figure S2. Recurrent copy number alteration identified byGISTIC2 in 4 dogs used in the analysis. A. Recurrent amplifications acrosscanine chromosome 1 - 38. A solid green line indicates the significancethreshold. B. Recurrent deletions across canine chromosome 1 - 38. A solidgreen line indicates significance threshold. Figure S3. Summary of somatic SNVs and Indels identified in the 7 canine tumors. A.Summary of somatic SNVs and Indels derived from maftools package, includingvariant classification, variant type, SNV class, number of variants in codingregion, and top 10 mutated genes. B. Somatic SNVs substitution type andtransition and transversion. Figure S4. Correlation between age at diagnosis and tumormutation burden (mutation per Mb). Figure S5. A. PCA plot of the 7 tumors based on geneexpression. B. Expression level of the GNAS gene in tumors with andwithout somatic GNAS mutation. Figure S6. Coverage of RNA-seq reads mapped to CNTN4(A), JAK3 (B), CATSPERE (C) gene for the 7 tumor samples. Table S1. Genotypes of the somatic GNAS mutation and germline TPO mutation in dogs. [file 12864_2022_8885_MOESM1_ESM.docx]

**Materials and Methods**

**Library construction and sequencing**

DNA samples were used for library construction following the manufacture’s recommendations using NEB Next® UltraTM DNA Library Prep Kit (Cat No. E7370L). Index codes were added to each sample. Briefly, the genomic DNA is randomly fragmented to an average size of 350 bp. DNA fragments were end polished, A-tailed, ligated with adapters, size selected, and further PCR enriched. Then PCR products were purified (AMPure XP system), followed by size distribution by Agilent 2100 Bioanalyzer (Agilent Technologies, CA, USA), and quantification using real-time PCR. Libraries were PE150 bp sequenced on a NovaSeq 6000 S4 flow cell.

**Somatic structural variants calling**

Only 4 pairs of samples (normal vs tumor) DogWUR108 vs DogWUR115, DogWUR112 vs DogWUR119, DogWUR113 vs DogWUR91 and DogWUR114 vs DogWUR120 were included in SV and CNA analyses. The other 3 pairs were not included due to the low and uneven coverage of matched normal WGS. A consensus approach was used to achieve a higher sensitivity and lower false discovery rate [1]. In this study, we used 4 somatic SV callers: GRIDSS [2], SvABA [3], Manta [4], and Delly [5]. All the callers were run using default settings. Then resulting SVs were filtered using the following criteria: SV length > 100 bp; variant supporting reads in normal sample = 0; mapping quality > 20. The filtered SVs were then merged using SURVIVOR [6] and SVs identified by 2 or more callers were retained. These consensus SVs also have to agree on the strand and have a distance of within 500 bp measured pairwise between breakpoints. Subsequently, a visual inspection was taken for each consensus SV using the Samplot package [7]. The SVs which are supported by paired reads and/or split reads present in the tumor but absent from the matched normal genome were flagged as true calls. Only the SVs passing the visual inspection were used for subsequent analyses. StructuralVariantAnnotation package [8] was used to annotate SVs, including identification of genes affected by SVs.

**Results**

### Somatic structural variants

To investigate the role of somatic structural variants (SV) in the tumorigenesis of the FCC in these GLPs, we used 4 methods to call somatic structural variants in paired tumor-normal mode and obtained the consensus somatic variants (reported by 2 or more callers), which passed a visual inspection using the Samplot package. The number of identified somatic SVs is diverse across samples (ranging from 6 to 32). The tumor sample DogWUR108 captured more somatic SVs than other dogs (Figure S1). Moreover, among conventional SV types (deletion, duplication, insertion, inversion, translocation), inter-chromosomal translocation was the most dominant type identified.

SVs can cause gene fusion thereby contributing to tumorigenesis. Gene fusions between *CNTN4* and two other genes were detected in 3 tumor samples (*CNTA4*/*JAK3* in DogWUR112, *CNTN4*/*CATSPERE* in DogWUR113 and DogWUR114). However, the expression of these fusion genes was almost 0 (Supplementary Figure S6 A-C), suggesting that these fusion events probably have limited consequence and do not contribute to tumorigenesis.

The consequences of other somatic SVs were also investigated in the forms of gene duplication, gene deletion, gene inversion, and gene structural interruption which was defined as the breakpoint of a somatic SV locating within the gene region. No common gene alteration across these 4 tumors was identified.


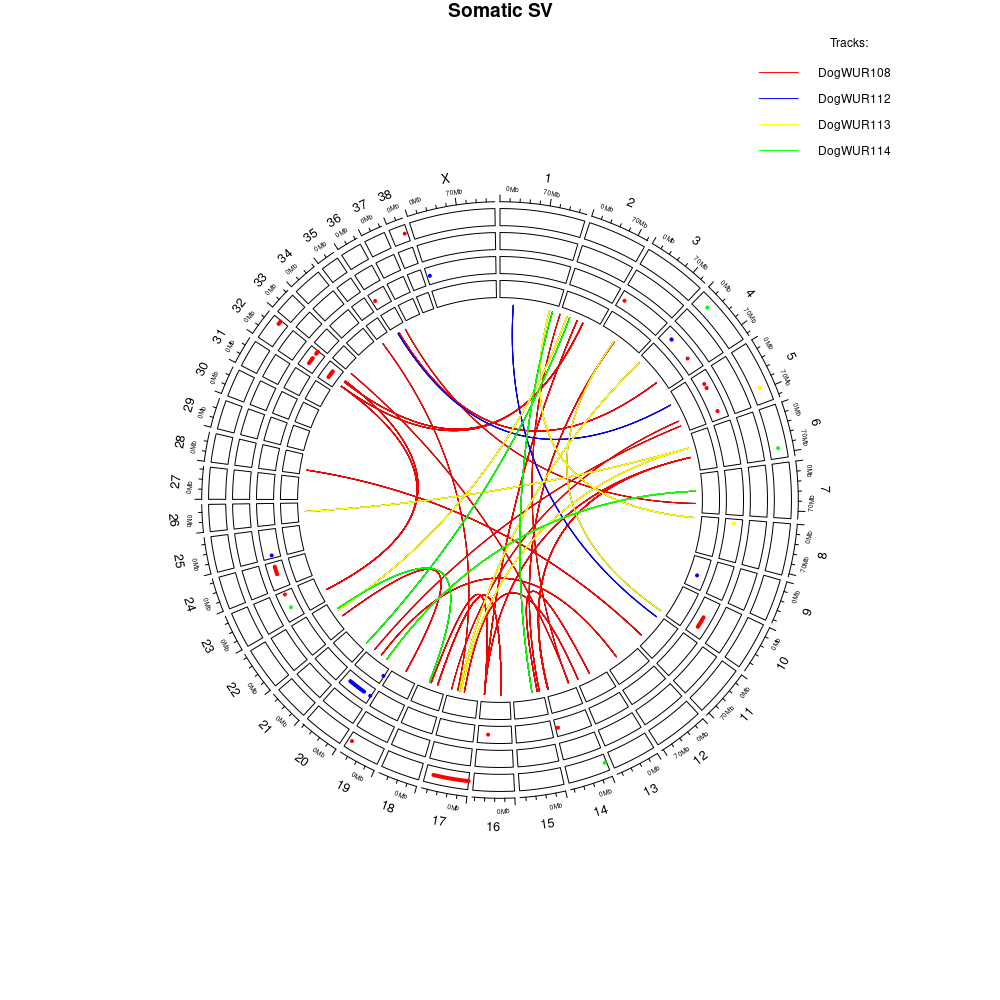


Figure S1. Landscape of somatic SVs identified in the tumors of 4 dogs. The circles represent deletion, insertion, inversion, duplication from the outside inwards respectively. The links inside represent the translocations. Different colors represent specific tumor sample, namely red - DogWUR108, blue - DogWUR112, yellow - DogWUR113, green - DogWUR114.

### Recurrent somatic CNAs

Recurrent CNAs could play an important role in carcinogenesis by altering the gene expression. Recurrent CNA was identified using the program GISTIC2 based on segmented data derived from the TitanCNA workflow. Three focal amplification peaks passing threshold were identified, which are on chr5, chr8, and chr37 respectively (Figure S2A). Many recurrent copy number deletions reached statistical significance, likely because of our small sample size (Figure S2B). We therefore decided to focus only on the recurrent amplifications in this study. In the 3 significant recurrent amplification regions, only 1 gene was located, which is the *ERBB4* gene on chr37. However, this gene was not expressed by analyzing the RNA-seq data. Therefore, this gene amplification likely did not contribute to tumorigenesis.

| 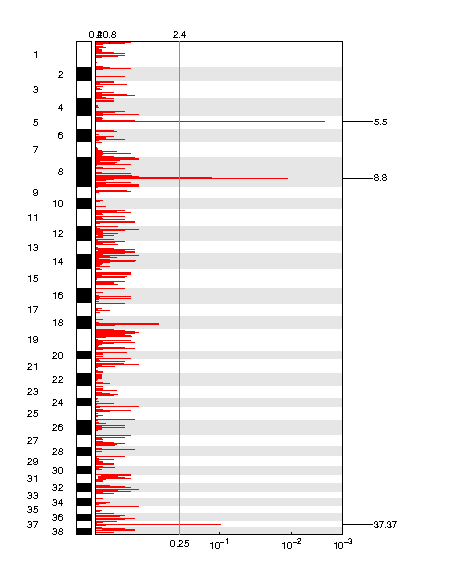 | 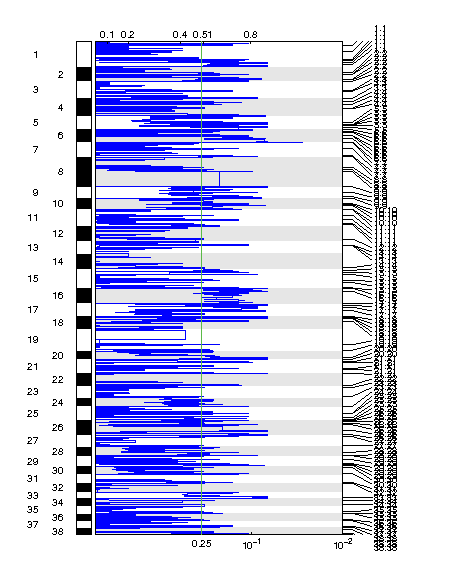 |
| --- | --- |
| A | B |

Figure S2. Recurrent copy number alteration identified by GISTIC2 in 4 dogs used in the analysis. A. Recurrent amplifications across canine chromosome 1 - 38. A solid green line indicates the significance threshold. B. Recurrent deletions across canine chromosome 1 - 38. A solid green line indicates significance threshold.

| A | B |
| --- | --- |
| 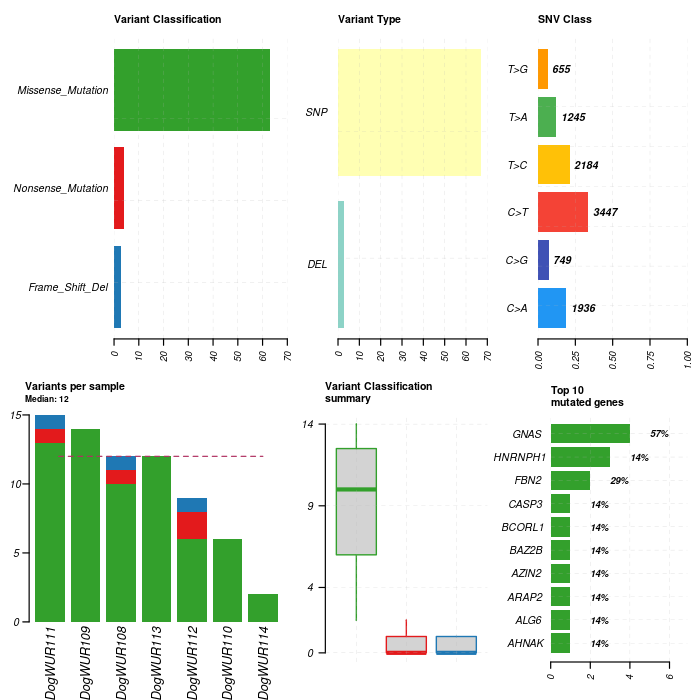 | 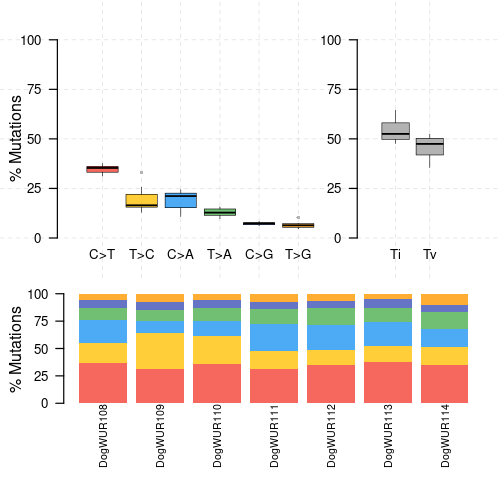 |

Figure S3. Summary of somatic SNVs and Indels identified in the 7 canine tumors. A. Summary of somatic SNVs and Indels derived from maftools package, including variant classification, variant type, SNV class, number of variants in coding region, and top 10 mutated genes. B. Somatic SNVs substitution type and transition and transversion.

Table S1. Genotypes of the somatic GNAS mutation and germline TPO mutation in dogs.

| Dog ID | Side of tumor on thyroid gland | GNAS | TPO |
| --- | --- | --- | --- |
| GLP184 | Left | - | - |
| GLP184 | Right | CA | - |
| GLP16 | Right | - | AA |
| GLP16 | Left | CC | AA |
| GLP75 | Right | CC | AG |
| GLP9 | - | CC | AG |
| GLP29 | Right | CA | AA |
| GLP29 | Left | CC | AA |
| GLP30 | - | CA | AG |
| GLP31 | Left | CA | AA |
| GLP31 | Right | CA | AA |
| GLP20 | Right | CA | AA |
| GLP20 | Left | CC | AA |
| GLP40 | - | CC | AA |
| GLP22 | Right | CA | AA |
| GLP22 | Left | CC | AA |
| GLP230 | Right | CC | AA |
| GLP230 | Left | CA | AA |
| GLP63 | Right | CA | AA |
| GLP63 | Left | CA | AA |
| GLP35 | - | CC | AA |
| GLP43 | - | CA | AA |
| GLP43 | Right | CC | AA |
| GLP15 | Right | CA | AA |
| GLP61 | - | CC | AG |
| GLP77 | Right | CC | AA |
| GLP77 | Left | CC | AA |
| GLP48 | Right | CC | AA |
| GLP48 | Left | CC | AA |
| GLP28 | - | CA | AG |
| GLP127 | - | CC | GG |
| GLP14 | Right | CC | AG |
| GLP44 | Left | CA | AA |
| GLP44 | Right | CA | AA |
| GLP68 | - | CA | AA |
| GLP17 | - | CA | AG |
| GLP7 | - | - | AA |
| GLP26 | Left | CA | AA |
| GLP25 | Left | CA | AA |
| GLP25 | Right | CA | AA |
| GLP34 | Right | CA | AA |
| GLP34 | Left | CA | AA |
| GLP21 | - | CC | AA |
| GLP24 | - | CA | AA |
| GLP18 | - | CC | AA |
| GLP4 | - | - | GG |
| GLP39 | Left | CA | AA |
| GLP39 | Right | CA | AA |
| GLP33 | Right | CA | AA |


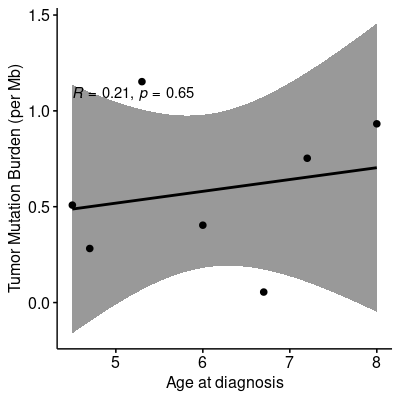


Figure S4. Correlation between age at diagnosis and tumor mutation burden (mutation per Mb).

| 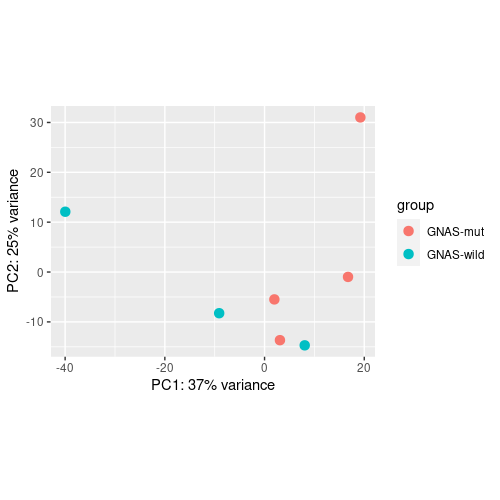 | 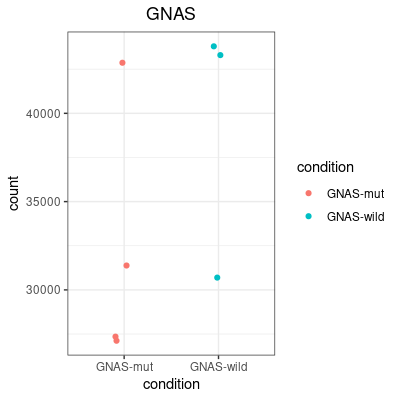 |
| --- | --- |
| A | B |

Figure S5. A. PCA plot of the 7 tumors based on gene expression. B. Expression level of the *GNAS* gene in tumors with and without somatic *GNAS* mutation.

| A | 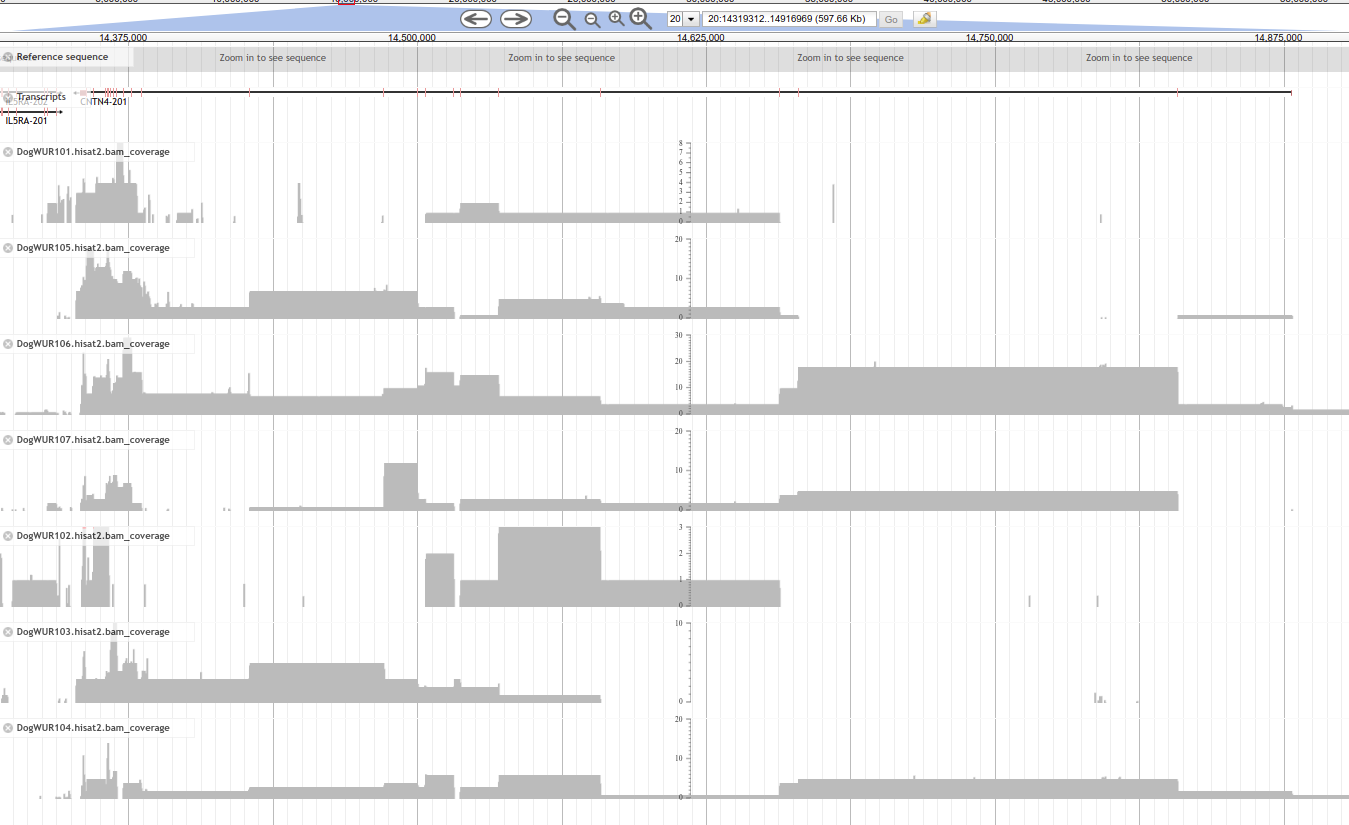 |
| --- | --- |
| B | 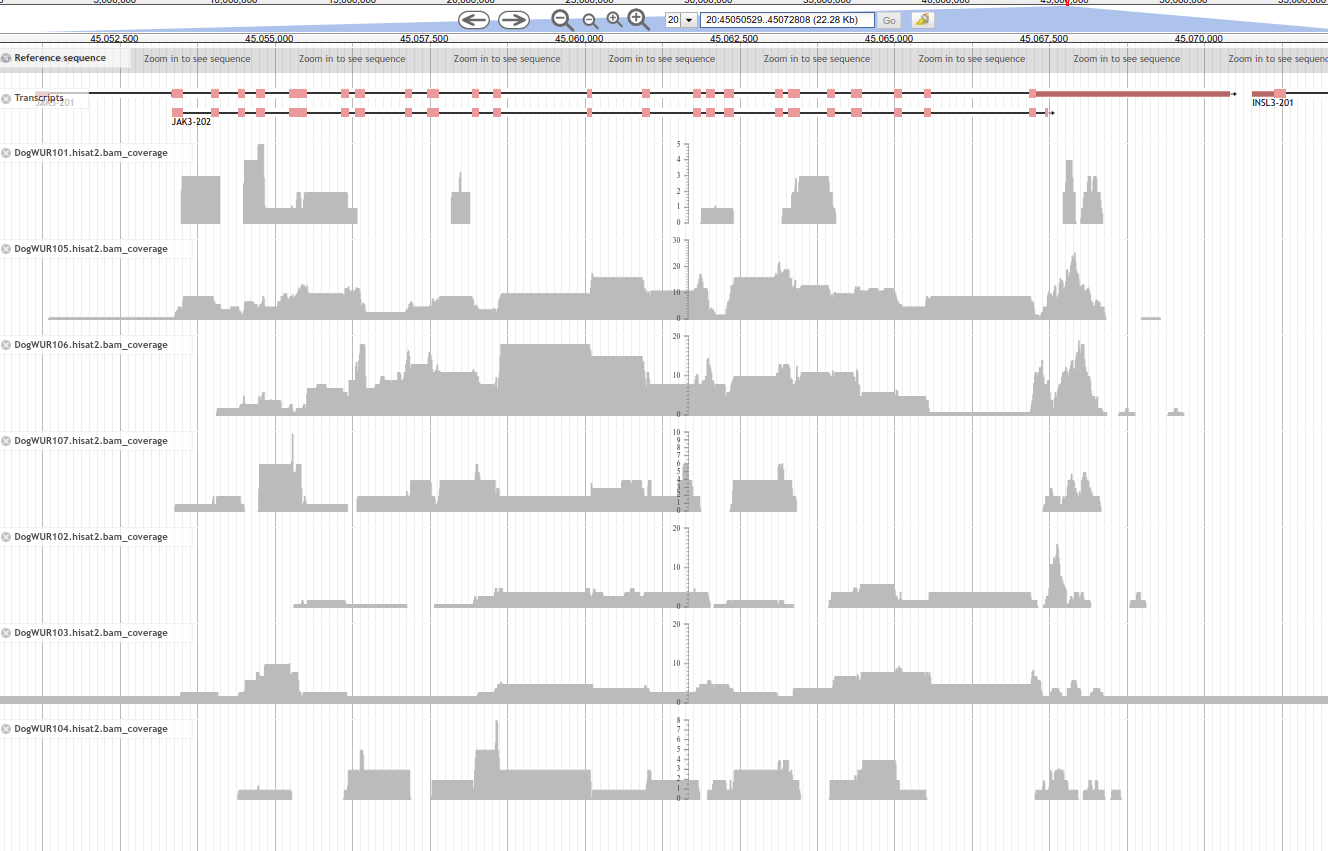 |
| C | 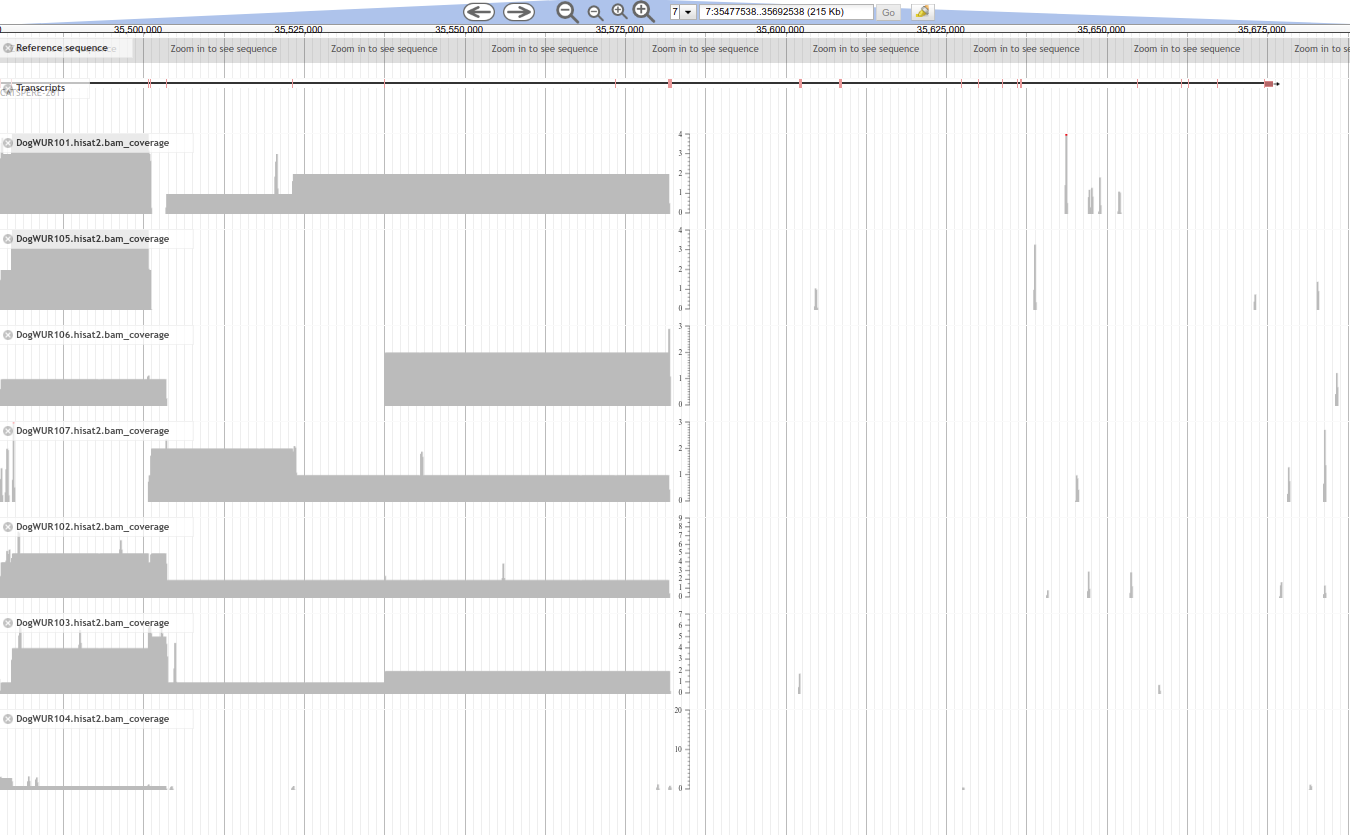 |

Figure S6. Coverage of RNA-seq reads mapped to *CNTN4* (A), *JAK3* (B), *CATSPERE* (C) gene for the 7 tumor samples.

**Reference**

1. Gong T, Hayes VM, Chan EKF. Detection of somatic structural variants from short-read next-generation sequencing data. Briefings in Bioinformatics. 2021;22(3).

2. Cameron DL, Schröder J, Penington JS, Do H, Molania R, Dobrovic A, et al. GRIDSS: sensitive and specific genomic rearrangement detection using positional de Bruijn graph assembly. Genome research. 2017;27(12):2050-60.

3. Wala JA, Bandopadhayay P, Greenwald NF, O'Rourke R, Sharpe T, Stewart C, et al. SvABA: genome-wide detection of structural variants and indels by local assembly. Genome Res. 2018;28(4):581-91.

4. Chen X, Schulz-Trieglaff O, Shaw R, Barnes B, Schlesinger F, Källberg M, et al. Manta: rapid detection of structural variants and indels for germline and cancer sequencing applications. Bioinformatics. 2016;32(8):1220-2.

5. Rausch T, Zichner T, Schlattl A, Stütz AM, Benes V, Korbel JO. DELLY: structural variant discovery by integrated paired-end and split-read analysis. Bioinformatics (Oxford, England). 2012;28(18):i333-i9.

6. Jeffares DC, Jolly C, Hoti M, Speed D, Shaw L, Rallis C, et al. Transient structural variations have strong effects on quantitative traits and reproductive isolation in fission yeast. Nature Communications. 2017;8(1):14061.

7. Belyeu JR, Chowdhury M, Brown J, Pedersen BS, Cormier MJ, Quinlan AR, et al. Samplot: a platform for structural variant visual validation and automated filtering. Genome Biology. 2021;22(1):161.

8. Cameron D DR. StructuralVariantAnnotation: Variant annotations for structural variants. R package version 1.8.1; 2021.
